# KRAS G12C Mutation Predicts Improved Survival in NSCLC Patients Receiving Immunotherapy: Insights from a Real-World Cohort

**DOI:** 10.3390/jcm14196826

**Published:** 2025-09-26

**Authors:** Aslı Geçgel, Buket Şahin Çelik, Pınar Peker, Zeynep Sıla Gökdere, Didem Koca, Burçak Karaca, Deniz Nart, Erdem Göker

**Affiliations:** 1 Department of Medical Oncology, Faculty of Medicine, Ege University, Kazım Dirik District, 35100 Izmir, Turkey; zeynep.sila.gokdere@ege.edu.tr (Z.S.G.); burcak.karaca@ege.edu.tr (B.K.); erdem.goker@ege.edu.tr (E.G.); 2 Department of Medical Oncology, Bursa Ali Osman Sönmez Oncology Hospital, 16040 Bursa, Turkey; shnbuket87@gmail.com; 3 Department of Medical Oncology, Adana City Hospital, 01370 Adana, Turkey; pinar.peker@saglik.gov.tr; 4 Department of İnternal Medicine, Faculty of Medicine, Ege University, 35100 Izmir, Turkey; didem.koca@ege.edu.tr; 5 Department of Medical Pathology, Faculty of Medicine, Ege University, 35100 Izmir, Turkey; deniz.nart@ege.edu.tr

**Keywords:** non-small cell lung cancer, KRAS G12C mutation, immune checkpoint inhibitor, overall survival

## Abstract

**Background:** KRAS mutations are among the most common oncogenic drivers in non-small cell lung cancer (NSCLC), with KRAS G12C emerging as a therapeutically targetable subtype. However, the prognostic relevance of KRAS G12C compared with non-G12C mutations in patients receiving immune checkpoint inhibitors (ICIs) remains unclear. **Methods:** We retrospectively analyzed 80 NSCLC patients treated with ICIs between January 2020 and July 2024; data were censored on 3 July 2025. The cohort included 32 KRAS-mutant (20 G12C, 12 non-G12C) and 48 KRAS wild-type patients. Clinicopathological features, treatment details, and survival outcomes were collected. Overall survival (OS) and progression-free survival (PFS) were estimated using the Kaplan–Meier method, with group comparisons made using the log-rank test. Univariate and multivariate Cox regression analyses were conducted to identify independent prognostic factors. **Results:** Among 80 NSCLC patients treated with ICIs, the median OS and PFS were 14.3 and 8.2 months, respectively. Survival outcomes were comparable between KRAS-mutant and wild-type patients. Within the KRAS-mutant subgroup (n = 32), baseline characteristics were generally balanced between G12C (n = 20) and non-G12C (n = 12) cases, with non-significant trends toward higher metastatic burden and PD-L1 ≥ 50% in the G12C group. Median OS was significantly longer in G12C patients than in non-G12C patients (20.7 vs. 6.4 months; *p* = 0.021), whereas PFS did not differ significantly (10.2 vs. 3.7 months; *p* = 0.181). In multivariate analysis, non-G12C mutation independently predicted increased mortality risk (HR 3.35, 95% CI 1.26–8.89; *p* = 0.015). For PFS, recurrent disease status was associated with improved outcomes in univariate analysis (HR 0.30, 95% CI 0.09–0.94; *p* = 0.040), but no independent predictors were identified in multivariate modeling. **Conclusions:** In NSCLC patients treated with ICIs, the KRAS G12C mutation was associated with significantly improved OS compared with other KRAS subtypes, independent of clinicopathological characteristics. These findings suggest distinct biological behavior of KRAS variants in immunotherapy response and warrant further prospective validation.

## 1. Introduction

Non-small cell lung cancer (NSCLC) constitutes approximately 85% of all lung cancer cases and remains a leading cause of cancer-related mortality worldwide [1]. Among molecular alterations, activating mutations in the Kirsten rat sarcoma viral oncogene homolog (KRAS) gene occur in approximately 25–30% of cases and constitute one of the most prevalent oncogenic drivers [2]. KRAS mutations are predominantly concentrated in lung adenocarcinomas and are uncommon in squamous histology, supporting the rationale to examine variant-specific effects under immune checkpoint inhibitor (ICI) therapy [3].

With the increasing complexity of lung cancer treatment, current guidelines recommend routine molecular profiling for actionable alterations, including EGFR, ALK, ROS1, BRAF, MET, RET, and NTRK, while also incorporating PD-L1 expression testing to guide ICI therapy [4,5]. The introduction of direct KRAS G12C inhibitors has further highlighted the need to clarify how KRAS subtypes interact with immunotherapy in routine practice.

KRAS mutations cluster mainly at codons 12 and 13, with G12C, G12D, and G12V being the most common [6]. Recent data indicate that KRAS subtypes are not biologically equivalent: G12C has been associated with higher PD-L1 expression and increased tumor mutational burden (TMB) across multiple cohorts, suggesting a more inflamed tumor microenvironment [7,8]. In contrast, mutations such as G12V and G12R have been associated with inferior outcomes in smaller retrospective cohorts [9]. Moreover, concurrent co-mutations (e.g., STK11, KEAP1, TP53) substantially influence prognosis and immunotherapy outcomes in KRAS-mutant NSCLC, underscoring the heterogeneity within this molecular subset [10,11]. Overall, prior studies report inconsistent ICI outcomes across KRAS subtypes, and robust real-world, allele-specific datasets remain limited [12,13]. Importantly, characterizing the interaction between KRAS subtypes and PD-L1 expression may provide predictive insights into ICI efficacy.

Given this context, our study evaluated the relationship between KRAS mutational subtypes, overall survival (OS), and PD-L1 expression in NSCLC patients treated with ICIs. Our aim is to determine whether allele-specific variants carry distinct prognostic or predictive value, thereby contributing to patient stratification and therapeutic optimization.

## 2. Materials and Methods

### 2.1. Study Population, Data Collection, and Endpoints

Between January 2020 and July 2024, 80 NSCLC patients who received ICI therapy at Ege University Faculty of Medicine Hospital, comprising 32 patients with KRAS mutations and 48 patients with KRAS wild-type status identified by next-generation sequencing (NGS), were included in the study, with data censored at the cutoff date of 3 July 2025. Data collected from hospital electronic medical records and patient charts included age, gender, number of chronic diseases, smoking status, ECOG performance status, date of diagnosis, diagnostic pathology results (histology), diagnosis status (de novo vs. recurrent metastatic), KRAS subtype, additional mutations detected by NGS, PD-L1 expression status and percentage level if positive, metastasis status and sites, line of ICI therapy (first-line vs. ≥second-line), type of ICIs received, ICI initiation and discontinuation dates, last follow-up date, and date of death if applicable.

All patients in this study received ICI therapy. Treatment regimens were categorized as either ICI monotherapy (pembrolizumab or nivolumab alone) or chemo-ICI (immunotherapy administered concurrently with chemotherapy). A small subgroup of patients treated with dual ICI (ipilimumab plus nivolumab) also received concurrent chemotherapy; therefore, these cases were classified under the chemo-ICI group rather than analyzed separately. For descriptive purposes, the distribution of individual agents (pembrolizumab, nivolumab, ipilimumab + nivolumab) is reported in the baseline characteristics, but survival analyses were performed using the clinically relevant grouping of mono-ICI versus chemo-ICI. This approach minimizes potential confounding from regimen heterogeneity and provides a clearer comparison of clinically relevant treatment strategies.

Demographic, clinical, and pathological data were retrospectively reviewed to evaluate therapeutic efficacy and perform survival analysis. The primary endpoint was overall survival (OS), defined as the time from the initiation of ICI therapy to death from any cause or last follow-up, to specifically capture ICI-related survival outcomes rather than survival from initial cancer diagnosis. The secondary endpoint was progression-free survival (PFS), defined as the time from the initiation of ICI therapy to documented radiological or clinical disease progression, or death from any cause, whichever occurred first. Patients without an event at the time of analysis were censored at the date of last disease assessment. Patients younger than 18 years were excluded. No other exclusion criteria were applied.

### 2.2. Molecular Profiling Workflow

Allele-specific subtypes and KRAS mutations were identified using clinically focused NGS tests. DNA extraction was performed using the AVENIO Tumor DNA Isolation and QC Kit (Roche Diagnostics, Basel, Switzerland), with quantification and quality assessment conducted on a Qubit 4.0 fluorometer. The same kit was used to determine the Q ratio as a measure of DNA quality prior to library preparation. Libraries were prepared according to the manufacturer’s instructions using the AVENIO Tumor Library Prep Kit V2 (Roche Diagnostics), incorporating unique molecular barcoding to mitigate duplicate reads associated with low-input, degraded FFPE DNA. Target enrichment was performed using the AVENIO Tumor Tissue Expanded Panel V2 (Roche Diagnostics, Basel, Switzerland), which covers 77 cancer-related genes, 6 fusion genes, and copy number variations for 3 cancer-related genes. Library size distribution was evaluated via on-chip electrophoresis (High Sensitivity DNA Screen Tape) using 1 µL of sample on an Agilent 4150 Tape Station System (Agilent Technologies, Santa Clara, CA, USA). Sequencing was performed on an Illumina NextSeq Dx 500 instrument (Illumina, San Diego, CA, USA) with a 2 × 150 bp High Output Flow Cell. Secondary analysis of raw sequencing data was carried out using Avenio Oncology Analysis Software (version 2.0.0; Roche Diagnostics, Basel, Switzerland), with variants, fusions, and CNVs detected through the Avenio Oncology Analysis Server. Tertiary analysis was conducted using Integrated Genomics Viewer v2.3.4 (IGV; Broad Institute, MIT & Harvard, Cambridge, MA, USA). Variants were classified according to ACMG, AMP, ASCO, and AACR guidelines. Additional databases, including COSMIC, TCGA, and ClinVar, were used to annotate cancer relevance and clinical significance via Navify Mutation Profiler Software (version 2.7; Roche Diagnostics, Basel, Switzerland).

### 2.3. Statistical Analysis

All statistical analyses were performed using IBM SPSS Statistics for Windows, Version 25.0 (IBM Corp., Armonk, NY, USA). Descriptive statistics were reported as frequencies and percentages for categorical variables, and as mean ± standard deviation (SD) and median (minimum–maximum) for continuous variables. OS and PFS were estimated using the Kaplan–Meier method, and differences between groups were assessed with the log-rank test. Associations between categorical variables were evaluated using the chi-square or Fisher’s exact test, as appropriate.

Univariate and multivariate Cox proportional hazards regression analyses were conducted to determine the impact of clinical variables on mortality and progression risk. Variables entered into the Cox models included age group (≤65 years or >65 years), diagnosis status (de novo or recurrent), number of comorbidities (0, 1, or ≥2), ECOG performance status (0–1 or ≥2), metastatic burden (liver or brain metastasis or ≥3 metastatic sites vs. other), KRAS mutation status (wild-type or mutant), KRAS subtype (G12C or non-G12C), PD-L1 expression level (negative, 1–49%, or ≥50%), and line of immunotherapy (first-line or ≥second-line). A *p*-value < 0.05 was considered statistically significant.

In addition to the overall cohort analysis, a predefined subgroup comparison was performed within the KRAS-mutant population to evaluate differences between patients harboring the G12C mutation and those with non-G12C KRAS mutations. Baseline demographic and clinical characteristics were compared between these subgroups using the chi-square or Fisher’s exact test for categorical variables and the Mann–Whitney U test for continuous variables. Survival outcomes (PFS and OS) were estimated separately for each subgroup using the Kaplan–Meier method, and differences were assessed with the log-rank test. Univariate and multivariate Cox regression analyses were conducted for both the overall cohort and the KRAS-mutant subgroup to identify factors independently associated with progression and mortality risk.

## 3. Results

The study included 80 NSCLC patients treated with ICIs, with a mean age of 62.5 ± 9.0 years; 61.3% were ≤65 years; and 78.8% were male. Most patients (77.5%) were active smokers, and 67.5% had at least one comorbidity. ECOG performance status was 0–1 in 83.8% of cases. At diagnosis, 88.8% presented with de novo metastatic disease.

Liver metastases were observed in 17.5% of patients, brain metastases in 30.0%, and high metastatic burden (liver or brain involvement, or ≥3 metastatic sites) in 53.8%. Adenocarcinoma histology predominated (87.5%). KRAS mutations were detected in 32 patients (40.0%), of whom 62.5% had G12C. PD-L1 expression was negative in 42.5%, 1–49% in 23.8%, and ≥50% in 33.8%. ICIs were administered as first-line therapy in 87.5% of cases, most commonly nivolumab (73.8%).

The median follow-up was 11.24 months (range: 0.90–110.93), with a mean of 16.06 ± 16.22 months. Disease progression occurred in 73.8% of patients, and 63.7% died during follow-up. Detailed baseline characteristics are presented in Table 1.

**Figure 1:**
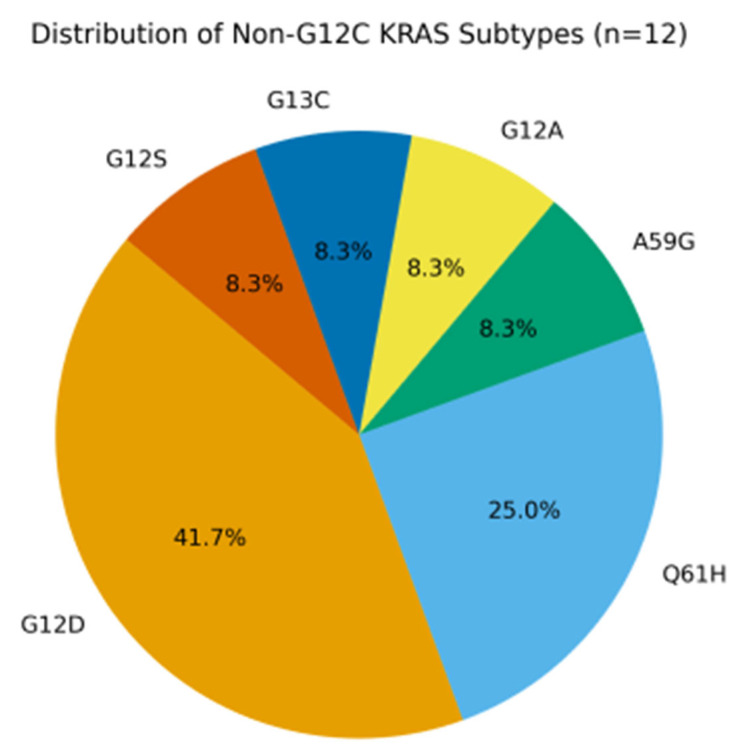
Distribution of non-G12C KRAS subtypes.

Within the KRAS-mutant cohort, baseline characteristics were generally balanced between patients with the G12C mutation (n = 20) and those with non-G12C mutations (n = 12) (Table 2). Trends were observed toward a higher metastatic burden (55.0% vs. 16.7%, *p* = 0.062) and higher PD-L1 expression ≥ 50% (50.0% vs. 25.0%, *p* = 0.081) in the G12C group, although these differences did not reach statistical significance and should be interpreted with caution. Smoking status and pack-year distribution were also comparable between the two groups. Among KRAS-mutant cases (n = 32), the non-G12C subgroup (n = 12) included G12D (n = 6), Q61H (n = 3), A59G (n = 1), G12A (n = 1), G12S (n = 1), and G13C (n = 1) (Figure 1).

The pie chart illustrates the distribution of non-G12C KRAS variants within our cohort (n = 12). The most common subtype was G12D (50.0%), followed by Q61H (25.0%), while G12A, G12S, G13C, and A59G were each observed in single cases (8.3% each).

In univariate analysis, diagnosis status was significantly associated with PFS (recurrent vs. de novo: HR = 0.29, 95% CI 0.09–0.94; *p* = 0.040). As no other variables met the inclusion criteria, no multivariable PFS model was fitted. For OS, both diagnosis status (recurrent vs. de novo: HR = 0.22, 95% CI 0.05–0.91; *p* = 0.037) and KRAS mutation subtype (non-G12C vs. G12C: HR = 2.82, 95% CI 1.13–7.06; *p* = 0.026) were significant in univariate analysis. After adjustment for covariates in the multivariate Cox model, non-G12C KRAS mutation remained an independent predictor of worse OS (HR = 3.35, 95% CI 1.26–8.89; *p* = 0.015), while recurrent disease showed only a non-significant trend toward improved survival (HR = 0.14, 95% CI 0.01–1.11; *p* = 0.063) (Table 3).

**Figure 2:**
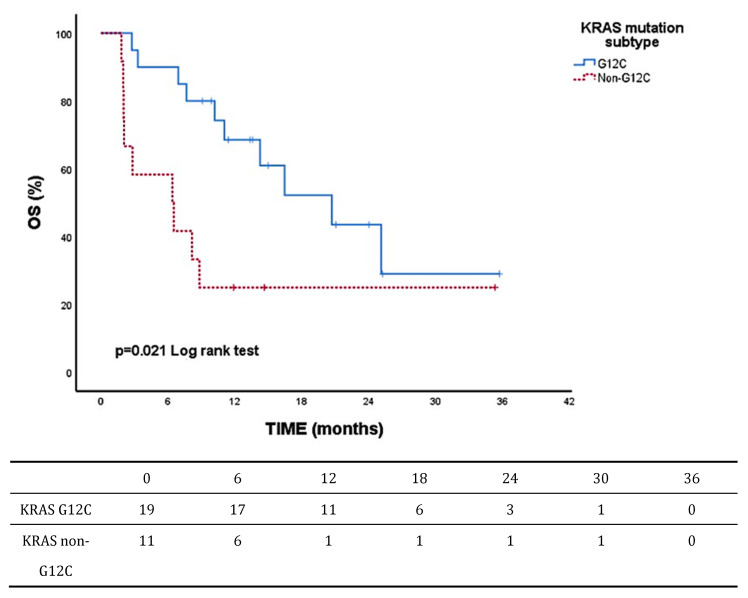
Kaplan–Meier OS curves according to KRAS mutation subtype. Kaplan–Meier; log-rank test; *p* < 0.05 considered statistically significant.

With a median follow-up of 11.24 months, the cohort’s median PFS was 8.16 months (95% CI, 3.77–12.56). The median OS was 14.26 months (95% CI, 5.41–23.11). By KRAS status (wild-type vs. mutant), OS (12.86 [0.15–25.57] vs. 14.26 [6.21–22.32]; *p* = 0.923) and PFS (8.30 [0.00–16.89] vs. 8.16 [1.37–14.96]; *p* = 0.792) were comparable. Within the KRAS-mutant subgroup, G12C appeared to have longer OS compared with non-G12C (20.70 [10.96–30.43] vs. 6.40 [0.12–12.68]; *p* = 0.021) and showed a non-significant trend toward longer PFS (10.20 [3.09–17.30] vs. 3.70 [1.77–5.62]; *p* = 0.181). Median OS and PFS values stratified by PD-L1 expression are provided in Table 4, with Kaplan–Meier curves shown in Figure 2. NR indicates not reached due to insufficient events or follow-up.

By PD-L1 category, median PFS was 4.93 months for PD-L1–negative patients, 16.20 months for those with PD-L1 1–49%, and 9.93 months for PD-L1 ≥ 50% (overall *p* = 0.146). Median OS was 8.83 months, not reached, and 16.46 months, respectively (overall *p* = 0.147); neither difference reached statistical significance.

In the KRAS-mutant subcohort (n = 32), the most frequent co-mutation was TP53 (21.9%; 7/32), followed by EGFR (9.4%; 3/32). Notably, one patient in this subgroup was also ALK-positive, representing a rare co-occurrence of two oncogenic drivers. Overall, 34.4% (11/32) of patients harbored at least one additional mutation, and 18.8% (6/32) carried two or more concurrent alterations. When stratified by subtype, the frequency of TP53 and EGFR mutations was comparable between G12C and non-G12C groups (22.2% vs. 21.4% and 11.1% vs. 7.1%, respectively). However, patients with G12C were more likely to exhibit multiple co-mutations (≥2 alterations: 22.2% vs. 7.1%) (Table 5).

In the KRAS G12C subgroup, three patients received sotorasib following chemo-immunotherapy, with PFS durations of 8.1, 15, and 9 months, respectively. Notably, one ALK-positive case, initially treated with brigatinib (PFS: 5 months) and subsequently with chemo-immunotherapy, also received third-line sotorasib, achieving a PFS of 8.1 months.

## 4. Discussion

### 4.1. Summary of Main Results

Our study showed that among KRAS-mutant NSCLC patients treated with ICIs, the KRAS G12C subtype was associated with a statistically significant and clinically meaningful OS advantage over non-G12C variants (median 20.7 vs. 6.4 months; *p* = 0.021). This effect persisted after adjustment (non-G12C: HR 3.35, 95% CI 1.26–8.89; *p* = 0.015). PFS favored G12C numerically but was not significant (*p* = 0.181). Additionally, when stratified by KRAS status (wild-type vs. mutant), OS (12.86 [0.15–25.57] vs. 14.26 [6.21–22.32]; *p* = 0.923) and PFS (8.30 [0.00–16.89] vs. 8.16 [1.37–14.96]; *p* = 0.792) were comparable.

### 4.2. Results in the Context of Published Literature

These findings are consistent with several real-world and multicenter reports. They indicate that the KRAS G12C mutation is associated with superior outcomes under ICI therapy [7]. However, this advantage has not been uniformly confirmed across all cohorts, as some studies reported inconsistent results [14,15,16]. In our series, PFS and OS were notably lower in the non-G12C subgroup. While this suggests biological differences among KRAS variants, the small sample size (n = 12), baseline imbalances, and treatment heterogeneity limit the robustness of these findings. Of note, some non-G12C patients received first-line single-agent ICI despite PD-L1 < 50%, an uncommon choice in clinical practice, likely reflecting advanced age and higher comorbidity burden. These factors may have reduced the feasibility of chemo-ICI and contributed to the poorer outcomes observed.

Collectively, evidence suggests that the favorable ICI responses in KRAS G12C may be driven by its mutational signature, tumor microenvironmental features, and co-mutation profiles [11,17,18]. TP53 co-mutations have been associated with enhanced immune activation. In contrast, STK11 and KEAP1 alterations contribute to ICI resistance. Importantly, resistance driven by STK11/KEAP1 may not be overcome by PD-1/PD-L1 blockade alone. This supports rational combinatorial strategies such as CTLA-4 inhibition [19]. In the first-line setting, consistent survival differences between G12C and non-G12C have not been demonstrated. By contrast, TP53 co-mutation has been linked to improved survival [20,21]. Some studies report that TP53 or STK11 co-mutations are not more frequent within the G12C subtype [22]. In our cohort, multiple co-mutations were observed, but at low frequencies. This precluded reliable subgroup analyses. To avoid unstable estimates, we did not model their effect on PFS or OS. Our findings should therefore be regarded as preliminary.

Within the KRAS-mutant cohort, TP53 (21.9%) and EGFR (9.4%) were the most frequent alterations; 34.4% of patients harbored ≥ 1 co-mutation, and 18.8% harbored ≥ 2. Multiple concurrent alterations were more common in the G12C group (TP53, n = 4; MET, n = 2; EGFR, n = 1), whereas in the non-G12C group, TP53 (n = 3), EGFR (n = 1), and BRCA1 (n = 1) were identified. Interestingly, we observed a KRAS-mutant case with an ALK rearrangement, a rare dual-driver event that has only sporadically been reported in the literature. Such findings raise important considerations for therapeutic prioritization and underscore the need for comprehensive genomic profiling. However, the modest sample size (particularly in the non-G12C subgroup) substantially limited the power to detect survival differences; as a result, analyses stratified by co-mutations or PD-L1 expressions could not be meaningfully performed. These descriptive findings highlight the need for validation in larger, multicenter cohorts with standardized genomic annotation and uniform biomarker assessment.

Importantly, the poor prognosis of the non-G12C subgroup cannot be explained by genetic differences alone. Lower PD-L1 expression and an immunologically “cold” tumor profile, together with STK11 and KEAP1 co-mutations, likely contribute to these unfavorable outcomes [23]. Larger series have demonstrated that STK11 and KEAP1 co-mutations, particularly in PD-L1–negative tumors, are associated with poorer prognosis [24,25,26]. Consistent with this, large-scale profiling datasets reveal heterogeneity in PD-L1 expression across KRAS subtypes, with G12C showing among the highest proportions of PD-L1 ≥ 50% [27]. In our cohort, PD-L1 ≥ 50% was more frequent in G12C compared with non-G12C, although this difference did not reach statistical significance (*p* = 0.081). The absence of survival differences across PD-L1 strata likely reflects the limited sample size and treatment heterogeneity (ICI monotherapy vs. chemo-ICI vs. sequencing). Nevertheless, PD-L1 should be recognized as an imperfect biomarker, and the lack of TMB data and immune microenvironment profiling precluded us from assessing the interplay between PD-L1, TMB, and other immune-related determinants of ICI response.

In addition to PD-L1, other immune evasion mechanisms warrant consideration. Loss of STK11/LKB1 suppresses type I interferon/STING signaling, reduces T-cell infiltration, and promotes a neutrophil- and myeloid-dominant microenvironment, conferring primary resistance to PD-1/PD-L1 blockade—even in PD-L1–positive tumors—and underscoring the limitations of PD-L1 as a standalone predictive biomarker [25]. Similarly, KEAP1/NRF2 alterations activate tumor-intrinsic antioxidant programs, impair dendritic and T-cell function, weaken antigen presentation, and contribute to poor prognosis. Emerging evidence underscores the pivotal role of KEAP1 in shaping ICI resistance and highlights the need for stratified therapeutic approaches in this subgroup [28].

Finally, KRAS G12C arises from G→T transversions induced by tobacco carcinogens and is strongly associated with a smoking-related mutational signature [2]. Large cohorts have demonstrated that KRAS G12C is more common in smokers, whereas KRAS G12D predominates in never-smokers [29]. This correlation is consistent with higher tumor mutational burden (TMB) and greater neoantigen diversity, features linked to a more inflamed tumor microenvironment and enhanced ICI sensitivity [29,30,31]. This smoking/TMB axis provides a biological rationale for the “antigenic richness” that facilitates T-cell recognition under PD-1/PD-L1 blockade [32]. In our series, smoking prevalence was similar across subgroups, suggesting that the observed differences are more likely attributable to PD-L1 expression and immune phenotype rather than smoking-related mutational load. Taken together, these findings underscore the need for future clinical trials to incorporate stratification by KRAS allele and co-mutation status, and to adopt multilayered biomarker models integrating PD-L1, TMB, co-mutations, and TME signatures to optimize ICI use in NSCLC.

In our cohort, recurrent metastatic disease was associated with more favorable outcomes compared with de novo presentation. In the multivariable model, recurrent disease independently predicted a lower risk of progression (HR 0.30, *p* = 0.047), with a concordant but not statistically significant trend for overall survival in sensitivity analyses (univariate *p* = 0.037; multivariable HR 0.14, *p* = 0.063). These findings are consistent with population-based observations showing that de novo metastatic NSCLC carries a poorer prognosis, plausibly reflecting earlier detection under surveillance, lower initial tumor burden, and potential biological differences in recurrent disease [33,34].

High metastatic burden was frequent in this series (53.8%), with liver (17.5%) and brain (30.0%) involvement being the most common sites. Notably, neither the composite burden metric (liver or brain involvement, or ≥3 sites) nor individual sites reached statistical significance in multivariable analyses for PFS or OS (e.g., brain: PFS HR 1.32, *p* = 0.308; OS HR 1.54, *p* = 0.137; liver: PFS HR 0.87, *p* = 0.719; OS HR 0.86, *p* = 0.707). This lack of association contrasts with prior reports indicating that liver involvement may attenuate ICI benefit, whereas clinically meaningful benefit remains achievable in selected patients with brain metastases, particularly with the judicious integration of local therapies [35,36]. Furthermore, in stage IV NSCLC, KRAS p.G12C is more frequent in women and more commonly associated with brain/CNS metastases (~28%); yet in this subgroup, brain involvement does not further worsen survival, while the expected adverse prognostic effect persists in KRAS-other (non-G12C mutations) and KRAS–wild-type cases [37]. The neutral results observed here are likely to reflect limited statistical power, treatment heterogeneity (ICI monotherapy vs. chemo-ICI), and selection effects. From an analytical perspective, subgroup evaluations (e.g., presence vs. absence of liver metastasis; brain metastasis with vs. without local therapy) and alternative burden definitions (e.g., M1b/M1c staging, or “liver plus ≥ 1 additional visceral site”) would help clarify site-specific biology and its interaction with immunotherapy.

In our cohort, treatment regimen (ICI monotherapy vs. chemo-ICI) was not associated with PFS in univariate analysis (*p* = 0.378), and because no univariate predictors met inclusion criteria, we did not fit a multivariable PFS model; only diagnosis status (recurrent vs. de novo) was significant in univariate analysis (HR 0.29, 95% CI 0.09–0.94; *p* = 0.040). Randomized trials of chemo-ICI backbones (e.g., KEYNOTE-189 and KEYNOTE-407) consistently demonstrate durable OS and PFS benefits across PD-L1 subgroups, whereas pembrolizumab monotherapy improves OS primarily in PD-L1–high populations (KEYNOTE-024; KEYNOTE-042) [38,39,40,41]. Beyond pembrolizumab, class-effect evidence with atezolizumab plus chemotherapy (e.g., IMpower150/130) also supports chemo-ICI benefit [42,43]; however, no patients received atezolizumab in our cohort, and these trials are cited for context rather than for direct comparison. The absence of a chemotherapy-only comparator and the small sizes of treatment subgroups in our series likely limited our ability to detect regimen-level differences. We therefore report regimen composition and indications (ICI monotherapy vs. chemo-ICI) in detail for transparency, while acknowledging potential confounding from treatment-regimen heterogeneity.

Overall, the prognostic role of KRAS subtypes remains inconsistent, likely due to heterogeneity in stage, patient characteristics, treatment line, molecular testing methods, and immune phenotype (PD-L1/TMB). KRAS subtypes may also differentially modulate downstream signaling, thereby influencing clinical behavior. These observations underscore the need for adequately powered studies that integrate PD-L1, TMB, and co-mutation status to clarify subtype-specific effects and support the rationale for routine panel-based KRAS testing, particularly for G12C.

### 4.3. Strengths and Weaknesses

This single-center, retrospective study provides real-world, allele-specific evidence on ICI outcomes in KRAS-mutant NSCLC, a setting where granular data remain limited. As with all retrospective, non-randomized studies, the risk of selection bias cannot be excluded, since patients receiving ICIs may differ from those who do not with respect to comorbidities, disease stage, or performance status. By stratifying KRAS mutations (G12C vs. non-G12C) and reporting Kaplan–Meier estimates alongside multivariable models, we offer clinically interpretable effect sizes while partially mitigating confounding.

Key limitations include the retrospective design; a modest sample size with NGS-based molecular data available only from 2020 to 2024, which restricts power—particularly for co-mutation–defined subgroups; treatment-regimen heterogeneity (ICI monotherapy, chemo-ICI); incomplete co-mutation (e.g., STK11/KEAP1/TP53) and TMB data; and potential temporal variability in PD-L1 assays. These factors could attenuate or exaggerate subgroup contrasts and may partly account for the observed OS advantage in G12C. Although 5-year OS and PFS rates were estimated using Kaplan–Meier methods, these results should be interpreted with caution, given the relatively short median follow-up of 11.24 months and the limited number of patients remaining at risk beyond 3 years.

Notably, to reduce measurement variability, all patients underwent NGS on a single platform, and PD-L1 scoring and KRAS subtyping were reviewed by the same clinician, strengthening internal consistency. Although the cohort size is modest, its clinicopathologic profile reflects routine practice; nevertheless, validation in larger, multicenter cohorts with standardized molecular annotation is warranted. Another limitation is the relatively short follow-up. The minimum potential follow-up for the last patient enrolled in July 2024 was approximately 12 months at the July 2025 data cut-off, whereas the overall median follow-up was 11.24 months. Future updates with extended follow-up and larger multicenter cohorts will be essential to validate these findings.

### 4.4. Clinical Implications and Future Directions

Our findings reinforce the importance of allele-specific biology: KRAS G12C tumors, often characterized by higher PD-L1 expression and TMB, may derive meaningful and durable benefit from ICIs in routine practice, whereas co-mutations such as STK11/KEAP1 can attenuate this benefit and should be incorporated into risk stratification and patient counseling. Treatment context is also critical; randomized trials have demonstrated that chemo-ICI backbones improve survival, particularly in PD-L1–low/negative disease, providing a plausible explanation for the heterogeneity in PFS observed across real-world strategies.

Looking ahead, prospective studies or well-annotated retrospective cohorts should systematically capture STK11/KEAP1/TP53 status, TMB, and smoking history. Metastatic burden and distribution (e.g., liver, brain), along with treatment sequencing and the use of local therapies, should also be documented. Such data will be essential for refining predictive models, given that liver metastases may blunt ICI benefit, whereas clinically meaningful outcomes are achievable in patients with brain metastases, particularly when local approaches are integrated. Finally, as KRAS G12C inhibitors (e.g., sotorasib and adagrasib) enter routine practice, studies addressing sequencing with ICIs are needed, including careful monitoring for hepatotoxicity when G12C inhibitors are administered after prior PD-(L)1 exposure.

The OS advantage observed in the G12C subgroup may have been partly influenced by the subsequent use of sotorasib in several patients. The integration of KRAS G12C inhibitors into treatment sequencing is increasingly relevant in clinical practice, and prospective studies are needed to clarify their impact when administered after ICI. Importantly, the development of targeted therapies for non-G12C alterations (e.g., KRAS G12D, G12V, Q61H) is also underway, with early-phase trials of selective inhibitors and pan-KRAS agents showing encouraging activity. These advances underscore that the survival gap between G12C and non-G12C subtypes may narrow as novel targeted therapies become available [10,44].

## 5. Conclusions

In this real-world cohort of ICI-treated NSCLC patients, KRAS G12C tended to show longer OS compared to non-G12C variants, whereas PFS differences did not reach statistical significance. Although baseline demographics and clinical features were broadly balanced between subgroups, the small sample size and heterogeneity of the non-G12C group limited the robustness of this finding. Taken together with prior evidence on the modifying role of co-mutations and PD-L1/TMB biology, our results should be considered preliminary and hypothesis-generating. These observations support the integration of expanded molecular profiling and treatment context when interpreting outcomes in KRAS-mutant patients and highlight the need for allele-specific validation in larger, multicenter cohorts.

## Figures and Tables

**Table 1:** Baseline demographic and clinical characteristics of the study cohort (n = 80).

Variable	N	%
**Age**		
Mean ± SD	62.48 ± 9.02	
Median (min–max)	63.0 (35–82)	
≤65 years	49	61.3
>65 years	31	38.8
**Gender**		
Female	17	21.3
Male	63	78.8
**Number of comorbidities**		
None	26	32.5
1	26	32.5
≥2	28	35.0
**Smoking status**		
No	18	22.5
Yes	62	77.5
**ECOG performance status**		
0	34	42.5
1	33	41.3
2	13	16.3
**Diagnosis status**		
De novo	71	88.8
Recurrent	9	11.3
**Liver metastasis**		
No	66	82.5
Yes	14	17.5
**Lung metastasis**		
No	25	31.3
Yes	55	68.8
**Lymph node metastasis**		
No	9	11.3
Yes	71	88.8
**Brain metastasis**		
No	56	70.0
Yes	24	30.0
**Bone metastasis**		
No	55	68.8
Yes	25	31.3
**Metastatic burden**		
Other	37	46.3
Liver or brain metastasis or ≥3 sites	43	53.8
**Number of metastases**		
≤2	40	50.0
≥3	40	50.0
**Histology**		
Adenocarcinoma	70	87.5
Squamous cell carcinoma	10	12.5
**KRAS status**		
Wild type	48	60.0
Mutant	32	40.0
**KRAS subtype**		
G12C	20	62.5
Non-G12C	12	37.5
**PD-L1 expression**		
Negative	34	42.5
1–49%	19	23.8
≥50%	27	33.8
**Line of immunotherapy**		
First-line	70	87.5
≥Second line	10	12.5
**Type of immunotherapy**		
Pembrolizumab	18	22.5
Nivolumab	59	73.8
Ipilimumab + nivolumab	3	3.8
**Immunotherapy regimen**		
Mono-ICI	62	77.5
Chemo-ICI	18	22.5
**Progression**		
No	21	26.3
Yes	59	73.8
**Mortality**		
Alive	29	36.3
Deceased	51	63.7
**Follow-up time (months)**		
Mean ± SD	16.06 ± 16.22	
Median (min–max)	11.24 (0.90–110.93)	

**Table 2:** Baseline Characteristics of KRAS-Mutant Patients by G12C Status.

Variable	G12C (n = 20)	Non-G12C (n = 12)	*p*-Value
Age group (≤65/>65)	13/7	7/5	0.724
Sex (F/M)	7/13	2/10	0.422
Comorbidities (0/1/≥2)	4/5/11	2/3/7	0.606
ECOG (0–1/≥2)	14/6	10/2	0.405
Diagnosis (de novo/recurrent)	16/4	11/1	0.631
Metastatic burden (high/other)	11/9	2/10	0.062
PD-L1 (neg/1–49/≥50)	9/1/10	5/4/3	0.081
Line of immunotherapy (1st/≥2nd)	12/8	8/4	1.000
Chemo IO/mono IO	6/14	3/9	1.000
Smoking status (yes/no)	14/6	9/3	1.000
Smoking pack-years (none/≤20/>20)	6/2/12	3/2/7	0.852

**Table 3:** Univariate analyses of PFS and OS, and multivariate analysis of OS using Cox regression according to clinical variables. A *p*-value < 0.05 was considered statistically significant.

	Univariate PFS		Univariate OS		Multivariate OS	
Variables	HR (95% CI)	*p*	HR (95% CI)	*p*	HR (95% CI)	*p*
Age ≤65 (Ref) vs. >65	0.87 (0.51–1.49)	0.626	0.88 (0.49–1.56)	0.666		
Sex Female (Ref) vs. Male	1.27 (0.64–2.52)	0.486	1.63 (0.76–3.47)	0.205		
Number of comorbidities		0.369		0.343		
None (Ref)						
1	1.35 (0.71–2.58)	0.365	1.48 (0.73–2.98)	0.266		
≥2	1.58 (0.83–3.01)	0.162	1.63 (0.82–3.25)	0.160		
Smoking status No (Ref) vs. Yes	0.97 (0.52–1.81)	0.939	0.88 (0.46–1.70)	0.723		
ECOG PS		0.688		0.655		
0–1 (Ref)						
	1.27 (0.73–2.23)	0.387	1.15 (0.63–2.09)	0.646		
≥2	1.14 (0.51–2.58)	0.739	1.46 (0.64–3.33)	0.363		
Diagnosis Denovo (Ref) vs. recurrent	0.29 (0.09–0.94)	0.040	0.22 (0.05–0.91)	0.037	0.14 (0.01–1.11)	0.063
Liver metastasis No (Ref) vs. Yes	0.87 (0.43–1.79)	0.719	0.86 (0.41–1.84)	0.707		
Lung metastasis No (Ref) vs. Yes	0.93 (0.53–1.62)	0.798	0.91 (0.51–1.66)	0.777		
LN metastasis No (Ref) vs. Yes	0.61 (0.28–1.27)	0.186	0.64 (0.28–1.43)	0.282		
Brain metastasis No (Ref) vs. Yes	1.32 (0.77–2.29)	0.308	1.54 (0.87–2.72)	0.137		
Bone metastasis No (Ref) vs. Yes	1.15 (0.66–2.01)	0.606	1.01 (0.55–1.85)	0.973		
Metastatic burden		0.731		0.428		
Other (Ref)						
Liver or Brain or ≥3 sites	1.09 (0.65–1.84)		1.25 (0.71–2.19)			
Number of metastatic sites ≤ 2 (Ref) vs. ≥3	0.89 (0.53–1.51)	0.688	0.98 (0.56–1.71)	0.952		
Histology Adeno (Ref) vs. SCC	1.84 (0.88–3.83)	0.101	1.52 (0.67–3.42)	0.308		
KRAS status Wild (Ref) vs. Mutant	1.07 (0.62–1.85)	0.792	1.02 (0.57–1.83)	0.923		
KRAS subtype G12C (Ref) vs. non-G12C	1.79 (0.75–4.29)	0.189	2.82 (1.13–7.06)	0.026	3.35 (1.26–8.89)	0.015
PD-L1 level		0.155		0.158		
Negative (Ref)						
1–49%	0.48 (0.23–1.01)	0.056	0.45 (0.21–1.02)	0.056		
≥50%	0.76 (0.43–1.36)	0.371	0.77 (0.42–1.42)	0.413		
Line of ICI 1st (Ref) vs. ≥2nd	1.15 (0.63–2.07)	0.642	1.31 (0.68–2.51)	0.415		
Chemo-ICI No (Ref) vs. yes	1.31 (0.71–2.40)	0.378	1.10 (0.56–2.14)	0.780		
Immunotherapy regimen						
Chemo-ICI (Ref) vs. Mono-ICI	0.76 (0.41–1.39)	0.378	0.90 (0.46–1.77)	0.780		

**Table 4:** Comparison of OS and PFS between patient groups. Kaplan–Meier curve, log-rank test; *p* < 0.05 considered statistically significant. Abbreviations: OS, overall survival; PFS, progression-free survival; NR, not reached. Only median survival values with 95% confidence intervals are reported due to the limited follow-up period.

Variables	Median OS (%95 CI)	*p*	Median PFS (%95 CI)	*p*
Overall	14.26 (5.41–23.11)		8.16 (3.77–12.56)	
KRAS				
Wild	12.86 (0.15–25.57)	0.923	8.30 (0.00–16.89)	0.792
Mutant	14.26 (6.21–22.32)		8.16 (1.37–14.96)	
Kras subtype				
G12c	20.70 (10.96–30.43)	0.021	10.20 (3.09–17.30)	0.181
Non G12c	6.40 (0.12–12.68)		3.70 (1.77–5.62)	
PD-L1 expression				
Negative	8.83 (0.00–18.99)	0.147	4.93 (1.36–8.51)	0.146
1–49%	NR		16.20 (0.00–52.33)	
>%50	16.46 (0.00–33.46)		9.93 (6.85–13.01)	

**Table 5:** Distribution of co-mutations in the KRAS-mutant cohort (n = 32).

Gene/Feature	Total n (%)	KRAS G12C (n = 20)	KRAS non-G12C (n = 12)
TP53	7 (21.9)	4	3
EGFR	3 (9.4)	1	2
ALK	1 (3.1)	1	0
STK11	0 (0.0)	0	0
KEAP1	–	–	–
MET	2 (6.3)	2	0
BRCA1	1 (3.1)	0	1
CTNNB1	1 (3.1)	1	0
FGFR2	1 (3.1)	1	0
≥1 co-mutation	11 (34.4)	7	4
≥2 co-mutations	6 (18.8)	5	1

## Data Availability

The data presented in this study are available on request from the corresponding author due to privacy and ethical restrictions.
